# The role of [^18^F]FDG-PET/CT in staging and treatment planning for volumetric modulated Rapidarc radiotherapy in cervical cancer: experience of the European Institute of Oncology, Milan, Italy

**DOI:** 10.3332/ecancer.2014.409

**Published:** 2014-03-05

**Authors:** Roberta Lazzari, Agnese Cecconi, Barbara A Jereczek-Fossa, Laura Lavinia Travaini, Veronica Dell’ Acqua, Federica Cattani, Stefania Rizzo, Cristiana Fodor, Fabio Landoni, Roberto Orecchia

**Affiliations:** 1 Advanced Radiotherapy Centre, European Institute of Oncology, Milan 20141, Italy; 2 Medical Physics Division, European Institute of Oncology, Milan 20141, Italy; 3 Department of Nuclear Medicine, European Institute of Oncology, Milan 20141, Italy; 4 Radiology Division, European Institute of Oncology, Milan 20141, Italy; 5 Cervical Cancer Centre, Division of Gynaecologic Cancer Surgery, European Institute of Oncology, Milan 20141, Italy; 6 University of Milan, Milan 20122, Italy

**Keywords:** cervical cancer, IMRT, Rapidarc radiotherapy, ([^18^F]FDG-PET/CT)

## Abstract

**Rationale:**

to evaluate the role of 18F-fluorodeoxyglucose positron emission tomography ([^18^F]FDG-PET) integrated with computer tomography (CT) scan [^18^F]FDG-PET/CT in the staging and target volume definition in Intensity Modulated RapidarcTM Delivery (RA-IMRT) in cervical cancer.

**Methods:**

From June 2010 to December 2011, 66 patients affected by cervical cancer, candidates for definitive or adjuvant radiochemotherapy, underwent standard staging with CT and magnetic resonance imaging (MRI). All patients underwent [^18^F]FDG-PET/CT in order to exclude distant metastases and to define gross tumor volume (GTV). 40 and 26 patients received exclusive and adjuvant radiotherapy, respectively. RA-IMRT with simultaneous integrated boost (SIB) to the positive disease technique was employed.

**Results:**

[^18^F]FDG-PET/CT has changed the stage, and radiotherapy treatment planning was modified in 25% and 7.7 % of patients that received definitive and adjuvant radiotherapy, respectively. Particularly [^18^F]FDG-PET/CT imaging showed metabolically active tumor in lymph nodes area, therefore the stage and the treatment planning changed for these patients.

**Conclusions:**

[^18^F]FDG-PET/CT leads to a better staging and definition of disease and has the potential of showing lymph-node metastasis not only within the pelvis but also in the para-aortic area. In addition, [^18^F]FDG-PET/CT is useful for better definition of the target volume and to produce a ‘dose painted’ treatment. This might also open the field for escalation dose regimens.

## Introduction

18F-fluorodeoxyglucose positron emission tomography ([^18^F]FDG-PET) integrated with computer tomography (CT) scan ([^18^F]FDG-PET/CT) plays an increasing role in the diagnosis and radiotherapy planning in gynaecological cancer, particularly for cervical cancer and especially when radiotherapy, with or without chemotherapy, is the primary treatment modality [1, 2].

For this reason, it is important to define clinical staging not only with conventional CT and magnetic resonance imaging (MRI), but also with metabolic imaging as [^18^F]FDG-PET/CT [3, 4].

As demonstrated in several retrospective studies, approximately 15–30% of newly clinically diagnosed cervical cancers have pelvic/para-aortic lymph-node involvement [5, 6]. An accurate staging of the disease at the time of diagnosis is mandatory to choose the appropriate therapeutic treatment. Clinical examination, tumour extension, tumour volume, lymph-node status, and parametrial invasion are pre-treatment prognostic factors, which, when positive, orientate patient treatment towards radiotherapy instead of surgery. Most of these factors can be detected by radiological staging.

The use of [^18^F]FDG-PET/CT is increasing in the staging and follow-up of patients with cervical cancer and suspected recurrent disease: there is evidence in the literature that [^18^F]FDG-PET/CT has a higher sensitivity than CT in depicting occult metastatic spread. In several papers, a relevant role in evaluating the entity of response to treatment and therefore to plan the subsequent therapeutic strategy has been suggested. MRI imaging is considered the best single imaging method for the determination of cervix tumour location and size; invasion into the parametria, pelvic side wall, or adjacent organs; and the presence of nodal enlargement. Surgical lymphadenectomy is still the reference standard in the diagnosis of nodal metastases, but it carries a risk of complications. Therefore, preoperative imaging assessment of nodes is important.

Recent studies demonstrate the high sensitivity and specificity of [^18^F]FDG-PET/CT to determine nodal metastasis, while MRI is the best imaging technique for primary tumour evaluation with an accuracy of 90% versus the 60% of CT scan [7]. The sensitivity of [^18^F]FDG-PET/CT for the pelvis and for the lymph-node area is 100% and 75%, respectively [7].

A meta-analysis of lymph-node detection by [^18^F]FDG-PET/CT and MRI in patients with cervical cancer demonstrated that the [^18^F]FDG-PET/CT was superior to CT and MRI, particularly for the lymph-node area [6, 7].

In this study, we described our experience using and comparing [^18^F]FDG-PET/CT with MRI and CT scans in pre-treatment staging to detect pathological lymph nodes and primary tumour extension or residual disease after surgery. In our institution, we started using [^18^F]FDG-PET/CT as a tool to better draw the target by fusing TC simulation images with those of the [^18^F]FDG-PET/CT. We realised that, in some cases, the positivity of [^18^F]FDG-PET/CT led to a modification of the staging of the disease.

We also analysed the subsequent changes in treatment planning and volumes. We assessed the validity of [^18^F]FDG-PET/CT to radiologically draw the target volume in intensity modulated Rapidarc delivery (RA-IMRT) with simultaneous integrated boost (SIB) in cervical cancer.

## Material and methods

### Study protocol

From June 2010 to December 2011, 66 patients affected by cervical cancer (Stage IIB–IVB) underwent [^18^F]FDG-PET/CT for tumour staging and definition of the gross tumour volumes (GTVs).

IMRT-SIB Rapidarc (RA; Varian Medical System, Palo Alto, California, United States) was implemented in our Radiotherapy Centre at the European Institute of Oncology, Milan, Italy. The Ethics Committee of the European Institute of Oncology has approved this perspective protocol (notify by European Institute of Oncology N.86/11). For all patients, the radiation treatment schedule also included a boost delivered with high- or pulsed-dose rate (HDR or PDR) brachytherapy.

## Inclusion criteria

Patients with cervical cancer who were candidates for definitive radiochemotherapy or adjuvant radio/chemoradiation were included in this study.

The patients were stratified into two groups:

- 40 patients who received curative exclusive treatment (group A);
- 26 patients who received post-operative treatment (group B).

The histological classification was squamous cell carcinoma (52 patients), adenocarcinoma (12 patients), and adenosquamous (two patients). The median age at the time of diagnosis was 49.8 years (range 29–89 years). In all but nine cases concomitant chemotherapy (weekly cisplatin 40 mg/mq) was administered during radiotherapy (Table 1).

## Imaging studies

All patients (group A and B) underwent pre-treatment clinical examination and standard imaging staging with a thorax–abdominal–pelvic CT scan; abdominal–pelvic MRI was performed in the patients receiving definitive treatment (group A). [^18^F]FDG-PET/CT was performed in all patients (group A and B).

## [^18^F]FDG-PET/CT

Whole-body (WB) imaging was carried out in all patients after a 50-min uptake period on a GE Discovery ST PET/CT scanner (GE Healthcare, Milwaukee, Wisconsin, United States) using a standard skull base to pelvis protocol. Image acquisition was performed with patients lying supine on the scanner bed with the arms along the body (in radiotherapy treatment position).

The CT acquisition protocol included a low-dose CT (120 kV, 80 mA, 0.8 s/rotation, pitch 1.35, 3.75-mm slice thickness) from the base of the skull to mid-thigh for attenuation correction followed by the WB PET scan (3 min per bed position). After the baseline acquisition, dedicated PET static emission images of the tumour region were acquired for the RT treatment planning. The PET scans were acquired in 3-D mode in a 256*256 matrix. Images were reconstructed by the Vue point attenuation weighted ordered-subset expectation maximisation algorithm (two iterations, 30 subsets) followed by a post-reconstruction smoothing Gaussian filter (4.5-mm full-width at half-maximum).

The image readout was performed on a Xeleris Workstation (GE Healthcare), which allows visualisation of [^18^F]FDG-PET, CT, and fused ([^18^F]FDG-PET/CT) sections in axial, coronal, and sagittal planes.

PET/CT images were interpreted by an experienced nuclear medicine physician, who had knowledge of all clinical and instrumental data, in collaboration with an experienced radiation oncologist.

The presence of pathological FDG uptake was indicated when tracer uptake was increased relative to uptake in surrounding tissue and normal structures, excluding physiological bowel, urinary activity, and non-specific genital uptake in young women.

In the case of pathological FDG uptake, its exact anatomic location was indicated on the basis of integrated CT findings.

## MRI technique

All MRI examinations were performed on a 1.5 T scanner (Avanto, Siemens, Erlangen, Germany). The routine abdomino–pelvic exam included: end breath-hold axial T2 and T1 sequences on the upper abdomen (slice thickness 4–5 mm); free-breathing axial T2-weighted sequences (slice thickness/gap: 5/1 mm; voxel size: 1.0 × 0.9 × 5.0 mm3; iPAT factor = 2); sagittal T2-weighted sequences (slice thickness: 4 mm; voxel size: 1.0 × 0.7 × 4.0 mm3; iPAT factor = 2); axial T1-weighted SE sequences (slice thickness/gap 5/1 mm; voxel size 1.0 × 0.9 × 5.0 mm3; iPAT factor = 2); and para-axial T2-weighted images, perpendicular to the longitudinal axis of the endocervical canal (slice thickness: 3 mm; voxel size: 1.0 × 0.9 × 3.0 mm3; iPAT factor = 2) on the pelvis.

When indicated, contrast medium (Gd-DTPA, 0.2 mmol/kg; flow rate = 2 mL/s) was administered intravenously and post-contrast series were acquired during the arterial, venous, and delayed phase on the upper abdomen, while a fat-saturated axial 3-D isotropic gradient-echo T1 sequence (TR/TE 7.0/2.1 ms; flip angle: 12º; 340-mm field of view; thickness/gap: 5/1 mm; voxel size: 0.9 × 0.9 × 0.9 mm3; no iPAT) was acquired on the pelvis.

## Radiotherapy techniques

### Volumes and contouring

After the positioning of two 2-mm silver seeds, useful as fiducial markers, into the cervical tumour or to the vaginal cuff, at the time of the simulation procedure, a CT scan was performed with adjacent 3 mm. Patients were scanned in supine position with a Combifix immobilisation device, with a mean bladder volume of 400 cm3 (range: 145–630 cm3). The same volume was obtained for all treatment days. The simulation CT scan was integrated and fused with [^18^F]FDG-PET/CT for all patients and also with MRI for patients who were candidates to receive definitive treatment for GTV delineation. The metabolic positive GTVs were defined as the gross extent tumour shown by CT and MRI imaging including positive lymph-node areas. The positive PET tumour and lymph nodes (PET)-GTV tumour (GTV-T) and (PET)-GTV-N were identified by a nuclear medicine specialist on the advantage windows workstation using a threshold level of 42% of the SUVmax.

The images were fused with CT simulation scan images. The radiation oncologist defined the site of the pelvic lymph nodes drawing the common, external and internal iliac, obturator vessels. The presacral lymph-node area, where no vessels could be seen, was identified from the specific anatomic area. PET-positive nodal disease was included in the volume definitions and the radiation oncologist defined the GTV from both the combined 18FDG-PET/CT scans and the CT simulation. MRI was used to help the radiation oncologists to define GTV-T for uterus, parametrial invasion, and ovarian localisation.

Tumour clinical target volume (CTV-T) and nodal clinical target volume (CTV-N) were created manually, giving to GTV-T margins identified by clinical criteria of microscopic spread and adding a 7-mm margin to GTV-N. The presacral lymph-node area was not expanded but directly added to a total CTV-N. Organs at risk (OAR) and all structures, such as muscles and bones, that were not microscopically infiltrated, if not supposed to be, were excluded from the expansion of CTV. The para-aortic lymph-node area was included in the radiotherapy treatment when there were positive [^18^F]FDG-PET/CT para-aortic lymph nodes and/or common iliac lymph nodes. The CTV para-aortic lymph node (CTV-PA) was created manually.

Margins of 15 mm in all directions and 10 mm in craniocaudal dimension were then added to CTV-T to create planning target volume (PTV-T); 5- and 7-mm expansions were given to obtain PTV-N and PTV-PA from CTV-N and CTV-PA, respectively.

For post-operative treatments, in which no GTV-T was supposed to be present, the volume of PTV was created expanding a CTV of 7 mm. The vaginal cuff CTV was drawn accurately considering clinical evaluation and histological findings. Usually, CTV was inferiorly extended by 3 cm of vagina, considered from the lowest visible vaginal seed, and superiorly for about 1–2.5 cm stopping to draw when intestinal loops were seen.

The OAR were drawn, and excluded from CTV but not from PTV.

## Dose prescription

The dose was prescribed to the PTV volume. Intensity modulated arc therapy with SIB technique was employed. The dose was prescribed to the PTV: 45–50.4 Gy (1.8 Gy/fraction) was prescribed to the T, N0 pelvic and/or para-aortic lymph nodes and 55 Gy (2.2 Gy/fraction) to the positive lymph nodes.

RA plans were generated using two coplanar arcs of 360° optimised simultaneously with a beam energy of 6 MV. The RA technique used continuous variation of the instantaneous dose rate, MLC leaf positions, and gantry rotational speed to optimise the dose distribution. The collimator angle was kept fixed and set to 20° for all patients.

The dose calculations and optimisations were performed using the Eclipse treatment planning system (version 8.6) for a Trilogy equipped with the Millennium multileaf collimator 120 leaves (leaf width at isocentre of 5 mm in the central 20-cm part of the field, 10 mm in the outer 2×10 cm). The maximal dose rate was set to 600 MU/min.

Dose calculation was performed with the AAA algorithm using a grid of 2.5 mm.

## Treatment verification

Based on the institutional setup verification protocols, for the first five days of treatment, a check of patients’ position was performed by means of kV cone beam CT system integrated in the machine.

Any variation of setup was then checked twice a week and the institutional action level protocols were applied.

For all patients, the radiation treatment schedule also included a boost delivered with HDR or PDR.

## Results

### Patients and imaging studies

At this time, all patients are in complete remission, there is particularly no evidence of local recurrence in the site of radiotherapy.

## Tumour status and stage (group A)

We did not find any difference in T stage in group A patients comparing CT, MRI, and [^18^F]FDG-PET/CT images but MRI played an important role in the definition of GTV-T, for a more accurate definition of parametrial invasion and ovarian localisation.

## Lymph-node status ad stage (group A)

In 10/40 patients (25%) who received exclusive treatment, [^18^F]FDG-PET/CT staging changed the stage when compared with conventional CT and MRI images of lymph-node status (N or M stage), particularly we found one positive external iliac lymph node in one patient (stage changed IIB versus IIBN1), and in nine patients, we found that para aortic lymph nodes were positive (M0 versus M1 = stage IVB).

## Status and stage (group B)

In 2/26 patients (7.7%) who received adjuvant treatment, [^18^F]FDG-PET/CT showed the persistence of disease: in one patient, in the T area (residual disease to the vaginal cuff) and in para-aortic area in the other patient.

These two patients received surgery in another institute in Italy.

Consequently, [^18^F]FDG-PET/CT staging changed the TNM and FIGO stage and the radiotherapy treatment planning in 25% and 7.7% of exclusive and adjuvant patients, respectively.

Table 2 shows the TNM and FIGO stage with CT and/or MRI and [^18^F]FDG-PET/CT and the change of the stage after [^18^F]FDG-PET/CT.

## Change in treatment planning

### Definitive treatment

**Figure 1.**
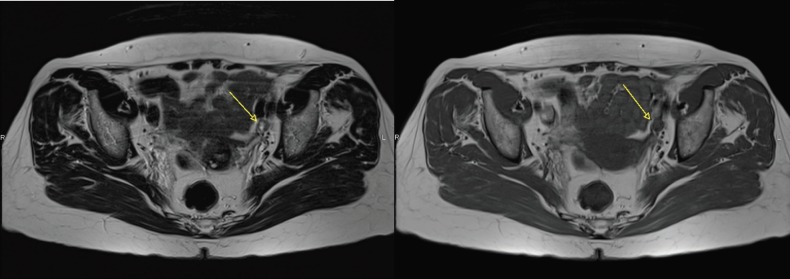
Axial T2 and T1 images showing a partially necrotic left external iliac node (arrow).

**Figure 2.**
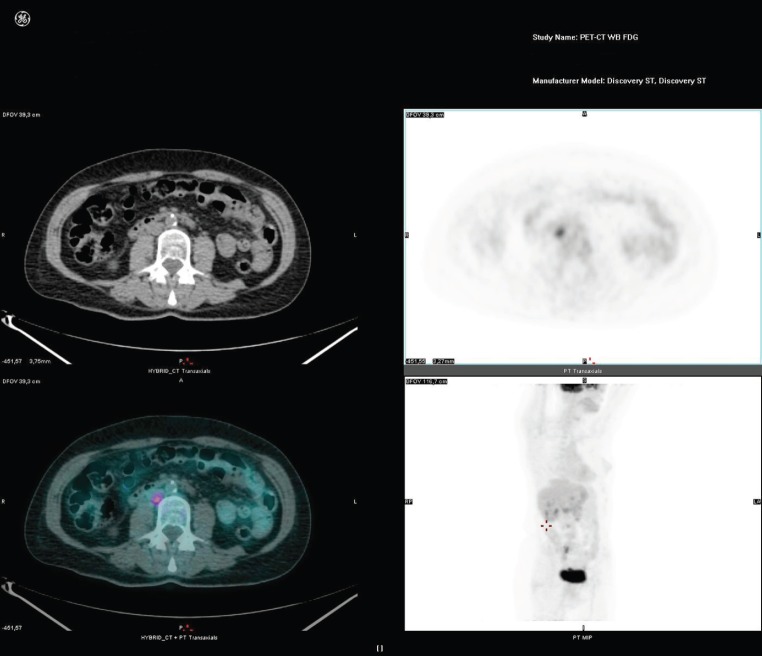
[^18^F]FDG-PET/CT has a higher sensitivity than CT in depicting lymph node metastatic.

In 10/40 patients (25%) who received definitive treatment, [^18^F]FDG-PET/CT has changed treatment planning. All patients received RA-IMRT delivered into the pelvis including the cervix tumour and pelvic lymph nodes. Ten patients where [^18^F]FDG-PET/CT changed the stage received RA-IMRT in the pelvis consisting in T area and negative pelvic lymph nodes (common, external and internal iliac, lymph nodes, obturator, and presacral lymph nodes) with SIB if there were positive lymph nodes (>1 cm): one patient received a SIB in the positive [^18^F]FDG-PET/CT iliac-external lymph node and four patients (one with positive [^18^F]FDG-PET/CT in common lymph node and three with positive para-aortic lymph node) received also RA-IMRT in the pelvis and para-aortic area with escalation of dose in positive lymph nodes (Figures 1 and 2).

## Adjuvant treatment

All patients received RA-IMRT in the pelvis: surgical bed (T and N-pelvic lymph nodes: included the common, external and internal iliac lymph nodes, obturator and presacral lymph nodes) and particularly one patient received a SIB in the positive T-([^18^F]FDG-PET/CT) area and one patient in the positive [^18^F]FDG-PET/CT para-aortic area.

## Discussion

The use of [^18^F]FDG-PET/CT is increasing in the staging and follow-up of patients with cervical cancer and suspected recurrent disease, as it is now clear from the literature that [^18^F]FDG-PET/CT has a higher sensitivity than CT in depicting occult metastatic spread and has an important role in therapeutic strategy [18–21].

MRI is considered the best single imaging method, with high accuracy of 88–97% and specificity of 93%, for the determination of tumour location and size, parametria invasion, pelvic side wall, and the presence of nodal enlargement [14].

Surgical lymphadenectomy is still the reference standard in the diagnosis of nodal metastases, but it carries a risk of complications. Therefore, preoperative imaging assessment of nodes is important.

The main criterion to define metastatic lymph nodes at MRI and CT is a short axis diameter >10 mm considering only the lymph-node size, sensibilities of CT and MRI are comparable, varying from 15% to 31% and 19% to 64%, respectively; while specificities range between 85–93% and 69–99%, respectively. The high number of false negatives is mainly explained by the high percentage of positive nodes measuring less than 10 mm in short axis diameter at pathological examination, and by the presence of microscopic metastases, below the detectable limits of imaging modalities. Thus, based on size criteria, both techniques have a limited value in the preoperative assessment of metastatic nodal spread.

Considering published studies including more than 30 patients, the sensitivity of PET/CT in evaluation of positive lymph nodes varies from 29% to 73%, and specificity varies between 84% and 97%, with a resolution of 5 mm. However, false-negative results by PET may occur due to small deposits of viable cancer cells (micrometastases) identified during histopathological analysis. On the other hand, large lymph nodes with no detectable tracer uptake deemed negative for metastases, but reactive changes resulted sometimes in false positives. Several articles have recently been published on the use of [^18^F]FDG-PET/CT for radiotherapy planning but no definitive conclusion could be drawn from these publications, with PET influencing target volume selection and delineation in some studies [5–7]. [^18^F]FDG-PET/CT has been increasingly used in the practice of radiation oncology for the assessment of tumour extension, thus leading to better staging, evaluation of treatment response, and recurrence.

Following the most recent guidelines, cervical cancer is diagnosed with clinical examination and imaging techniques, such as MRI and CT scan [6].

A large number of publications have demonstrated that the traditional MRI imaging is superior to CT for tumour delineation in cervical cancer [8, 9] but recently some publications have highlighted the utility of [^18^F]FDG-PET/CT for treatment planning guide, particularly for nodal delineation [10].

The first aim of our study was to detect possible discrepancies between [^18^F]FDG-PET/CT, MRI, and CT scans in pre-treatment staging to detect pathological lymph nodes and primary tumour extension or residual disease after surgery. In our study, 66 consecutive patients underwent both [^18^F]FDG-PET/CT, CT and MRI (only the patients of group A). We showed that, after the [^18^F]FDG-PET/CT, the stage was changed in 25% of group A and 7.7% in group B patients, respectively.

Kidd *et al* [11] analysed a group of 560 patients and found that [^18^F]FDG-PET/CT was more sensitive than CT scan for lymph-node staging in patients with cervical cancer. The authors concluded that 47% of the patients had lymph-node involvement by [^18^F]FDG-PET/CT at diagnosis. For lymph-node tumours larger than 5 mm, [^18^F]FDG-PET/CT had a sensitivity of 100%.

[^18^F]FDG-PET/CT effectively identifies lymph-node metastasis before any treatment has been initiated, identifying patients with a high risk of recurrence and patients who can benefit from a personalised treatment.

Several studies have shown that the possibility to extended field irradiation for treatment of patients with para-aortic lymph-node metastasis is feasible with [^18^F]FDG-PET/CT [11].

Loft *et al* [12] investigated the clinical value of [^18^F]FDG-PET/CT as a supplement to the FIGO staging system in patients with cervical cancer stage >IB. The prospective study included 120 consecutive patients. The [^18^F]FDG-PET/CT was performed after standard staging. The treatment results were compared with histopathological findings. Twenty-seven patients underwent radical surgery, of whom four had positive [^18^F]FDG-PET/CT in the pelvis. Three (11%) were true positive and one false positive. Twenty-two patients had true negative pelvic lymph nodes, and one patient had a false-negative node. [^18^F]FDG-PET/CT had a positive predictive value of 75% and negative predictive value of 96%, with a sensitivity of 75% and specificity of 96%.

The second aim of our study was to investigate the subsequent changes after [^18^F]FDG-PET/CT in radiotherapy treatment planning and volumes. In our analysis, [^18^F]FDG-PET/CT has changed the radiotherapy treatment volume in 25% and 7.7% of patients who received exclusive and adjuvant radiotherapy, respectively.

Exact identification of pre-treatment lymph-node status with [^18^F]FDG-PET/CT will be important not only for stage, but also to deliver additional treatment in the positive area.

Esthappan *et al* [5] used [^18^F]FDG-PET/CT as a guide of IMRT for escalation dose of positive para-aortic lymph nodes.

The patients with high-risk cervical cancer have a risk of positive para-aortic nodes ranging from 15% to 30%; to define positive disease in the para-aortic lymph-node, CT and MRI have a sensitivity of 30%. [^18^F]FDG-PET/CT seems to offer a very pertinent examination to define the extension of disease with 8–13% of false negative [13].

In a series of 32 patients with advanced-stage cervical cancer, Rose *et al* [15] reported PET sensitivity and specificity of 75% and 92%, respectively. Sensitivity was higher for pelvic (100%) than for para-aortic (75%) lymph-node metastases. Therefore, limited information of comparison between [^18^F]FDG-PET/CT and histological evaluation is available. In our study, no comparison between [^18^F]FDG-PET/CT data and surgical staging was performed. We believe that this information should be an object of future study.

Uzan *et al* [13] affirm that the CT, MR, and PET are not efficient enough to detect para-aortic disease; surgical staging is useful to detect associated carcinosis and adapt therapy.

It is important to remember that the surgical staging of patients with locally advanced cervical cancer remains controversial.

Ramirez *et al* [16] compared [^18^F]FDG-PET/CT with laparoscopic extraperitoneal surgical staging in the evaluation of para-aortic lymph nodes in 65 patients. The results suggest that this procedure should be discussed with patients with locally advanced cervical cancer to undergo chemoradiation, particularly if preoperative [^18^F]FDG-PET/CT shows positive pelvic nodes and negative para-aortic nodes.

Tsai *et al* [17] evaluated the utility of [^18^F]FDG-PET in determining the appropriate treatment field for cervical cancer patients with enlarged pelvic nodes on CT or MRI. They found that PET findings modified treatment in 28% of patients.

Yildirim *et al* [19] analysed 16 patients with stage IIB–IVA cervical cancer who had no evidence of disease in the lymph-node area on conventional CT scan and underwent [^18^F]FDG-PET/CT followed by surgery. The authors reported that the sensitivity and specificity of [^18^F] FDG-PET/CT in detecting metastasis to the para-aortic nodes were 75% and 50%, respectively. Staging and then treatment were modified on [^18^F]FDG-PET/CT in 25% of patients.

[^18^F]FDG-PET/CT improves accuracy of staging reducing possible confounding factor in radiotherapy treatment planning guide. Most of the changes resulted from the additional information on nodal involvement.

The utilisation of surgery for objective histological evaluation has not yet been fully validated. Therefore, the utilisation of such techniques for objective tumour evaluation should be restricted to validation purpose in future study of our Cervical Cancer Centre.

The main aim of our study was to evaluate the role of [^18^F]FDG-PET/CT in the staging and target volume definition in cervical cancer patients. In our experience, [^18^F]FDG-PET/CT has changed the stage and the radiotherapy treatment volume in 25% and 7.7% of patients who received exclusive and adjuvant radiotherapy, respectively. Therefore, all implications can only be suggested but not demonstrated with histological evaluation, and this represents the main limit of our study.

## Conclusions

[^18^F]FDG-PET/CT leads to a better staging of disease and a better volume definition, for unresectable cases, particularly for lymph-node metastasis in the pelvis and para-aortic area. Using [^18^F]FDG-PET/CT, it was possible to better define the target volume producing a ‘dose-painted’ treatment especially performing a SIB-IMRT arc therapy. A better target definition obtained by fusing [^18^F]FDG-PET/CT with simulation CT scan imaging leads to the possibility of delivering higher doses to small volumes so that dose escalation protocols can be conceived.

## Figures and Tables

**Table 1.** Patient characteristics.

		Number of Patients	%
Site	Cervix	66	
Histology	Squamous cell carcinoma Adenocarcinoma Adenosquamous	52 12 2	78.8 18.2 3
Age (years)	Median age (range)	49.8 (29–89)	
Diagnostic imaging	CT MRI ([^18^F]FDG-PET/CT)	66 40 66	100 60.6 100
Type of treatment	Exclusive treatment Adjuvant treatment	40 26	60.6 39.4
Chemotherapy	Yes No	57 9	86.4 13.6

CT: computer tomography.

MRI: magnetic resonance imaging.

[^18^F]FDG-PET/CT: 18F-fluorodeoxyglucose positron emission tomography integrated to computer tomography.

**Table 2.** TNM and FIGO stage with CT, MRI, and [^18^F]FDG-PET/CT and the change of the stage after [^18^F]FDG-PET/CT.

N	CT and MRI Stage (TNM-FIGO)		([^18^F]FDG-PET/CT) Stage (TNM-FIGO)	
1	T2bN0M0	IIB	*T2bN1M0*	*IIBN1*
2	T1bN1M0	IIBN1	*T1bN1M1*	*IVB*
3	T2bN0M0	IIB	*T3BN0M1*	*IVB*
4	T3aN1M0	IIBN1	*T3aN1M1*	*IVB*
5	T1bN1M0	IIBN1	*T1bN1M1*	*IVB*
6	T2aN0M0	IIA	*T2aN1M1*	*IVB*
7	T2bN1M0	IIBN1	*T2bN1M1*	*IVB*
8	T3aN1M0	IIIAN1	*T3aN1M1*	*IVB*
9	T2bN1M0	IIBN1	*T2bN1M1*	*IVB*
10	T1bN0M0	IB2	*T1bN1M1*	*IVB*
11	pT2bpN0M0	IIB	*pT2bpN0M0*	IIB^*^
12	pT2apN1M0	IIBN1	*pT2bpN1M1*	*IVB*

CT: computer tomography.

MRI: magnetic resonance imaging.

[^18^F]FDG-PET/CT: 18F-fluorodeoxyglucose positron emission tomography integrated to computer tomography.

([^18^F]FDG-PET/CT) staging changed the TNM and FIGO stage in 12/66 (18.2%) patients.
